# Clinical application research of brain natriuretic peptide in patients with aneurysmal subarachnoid hemorrhage

**DOI:** 10.1097/MD.0000000000044985

**Published:** 2025-10-10

**Authors:** Aiying Guo, Cuijie Ye, Jing Li, Wei Cao

**Affiliations:** a Department of Critical Care Medicine, Beijing Tiantan Hospital, Capital Medical University, Beijing, China.

**Keywords:** aneurysmal subarachnoid hemorrhage, brain natriuretic peptide, major adverse cardiac event, prognosis

## Abstract

This study investigated the relationship between increased brain natriuretic peptide (BNP) levels following aneurysm rupture and the incidence of major adverse cardiac events (MACE), perioperative complications, and long-term neurological outcomes. We included patients with aneurysmal subarachnoid hemorrhage within 72 hours of bleeding, collecting comprehensive clinical data. Participants were divided into 2 groups based on initial serum BNP levels: elevated (>100 pg/mL) and non-elevated (≤100 pg/mL). To mitigate bias, propensity score matching was employed to balance statistically significant variables. Follow-up assessments focused on in-hospital complications, MACE, and neurological outcomes. A total of 213 patients were analyzed, with an average age of 57.4 ± 12.1 years; 61.5% were women. The average follow-up duration was 34.3 months, with hypertension and coronary heart disease as common histories. After propensity score matching, the elevated BNP group showed higher rates of myocardial infarction (13.3% vs 4.0%, *P* = .001), acute heart failure (25.3% vs 4.0%, *P* = .042), delayed cerebral ischemia (25.3% vs 10.7%, *P* = .032), and hydrocephalus (13.3% vs 1.3%, *P* = .009). However, there was no significant difference in MACE between groups during follow-up. Logistic regression identified admission BNP as an independent risk factor for MACE (OR: 4.029, 95% CI: 1.753–9.262, *P* = .001), myocardial infarction (OR: 4.405, *P* = .038), heart failure (OR: 8.420, *P* = .002), delayed cerebral ischemia (OR: 3.449, *P* = .013), and hydrocephalus (OR: 13.726, *P* = .020). Elevated BNP levels after intracranial aneurysm rupture are associated with MACE, delayed cerebral ischemia, and hydrocephalus during hospitalization, but not with neurological status or MACE at discharge and follow-up.

## 1. Introduction

Nontraumatic cerebrovascular events caused by ruptured intracranial aneurysms are known as aneurysmal subarachnoid hemorrhage (aSAH), accounting for approximately 75% to 80% of nontraumatic subarachnoid hemorrhages. In 2019, the number of aSAH cases in China was 2,20,000, with an incidence rate of 15.6 per 1,000,000, representing a 35.6% increase compared to 1990, and a 54.8% increase in incidence rate compared to 1990.^[1]^ Subarachnoid hemorrhage constitutes 5.5% to 9.7% of all types of strokes.^[1,2]^

Aneurysmal subarachnoid hemorrhage occurs at a relatively young age (average of 55 years) and has a poor prognosis, with a mortality rate ranging from 32% to 67%. Among all surviving patients, 10% to 15% are left with functional dependence.^[3–6]^ In addition to previously recognized prognostic factors such as delayed cerebral ischemia (DCI) and acute hydrocephalus, other clinically relevant issues affecting prognosis after SAH include concomitant cardiac abnormalities following the onset or progression of SAH. The delicate balance of brain-heart interaction is disrupted after aSAH.^[7–10]^

Previous studies have shown that cardiac biomarkers, including creatine kinase-MB and troponin, are associated with aSAH-related DCI, in-hospital mortality, and short-term adverse neurological outcomes.^[11–14]^ Among these cardiac biomarkers, elevated levels of brain natriuretic peptide (BNP) have been reported in 19% to 39% of emergency department admissions for aSAH.^[15,16]^

The clinical prognostic value of elevated BNP levels in the acute phase of aSAH is still unclear. This study aims to investigate whether BNP levels upon admission in patients with acute aSAH are associated with in-hospital complications, long-term neurological functional outcomes, and major adverse cardiac events (MACE).

## 2. Materials and methods

### 2.1. Population

During the period from January 2016 to December 2017, a consecutive admission of 213 patients with aSAH who underwent myocardial enzyme laboratory testing within 72 hours after rupture was conducted at Beijing Tiantan Hospital, affiliated with Capital Medical University. The diagnosis of aSAH was based on the 2012 American Heart Association guidelines.^[17]^ This study complied with the Helsinki Declaration and was approved by the local Ethics Committee (Approval No. KY2021-00801).

Inclusion criteria were as follows: patients diagnosed with aSAH based on CTA or DSA with a hunt-hess grade of 1 to 5; initial presentation at our hospital; myocardial enzyme testing performed within 72 hours after aneurysm rupture; complete clinical data and information; informed consent provided by the patient or authorized representative. Exclusion criteria were as follows: concomitant presence of other intracranial malignant diseases, such as cerebral arteriovenous malformation or intracranial malignant space-occupying lesions; subarachnoid hemorrhage caused by other etiologies, such as venous sinus thrombosis or Moyamoya disease; incomplete clinical data.

### 2.2. Parameters

Clinical data of the patients, including demographic information (age, gender), smoking history, alcohol consumption history, medical history (including diabetes, hypertension, dyslipidemia, history of stroke, etc), history of cardiovascular diseases (including coronary heart disease, heart failure, arrhythmias, etc), history of brain surgery, and history of cardiac surgery, were collected from the electronic medical record system of the hospital. The modified Rankin Scale (mRS) was used to assess the neurological functional status of the patients, and the Hunt-Hess scale was used to evaluate the clinical severity of aSAH. Radiological features included the Fisher score and the presence of intraventricular hemorrhage. The main treatment modalities for ruptured aneurysms included surgical clipping, endovascular treatment, and conservative medical management.

The first serum BNP level within 3 days after aneurysm rupture was selected as the grouping variable, and was obtained prior to surgical clipping or endovascular treatment. Based on the results of BNP measurements using the BNP assay kit (ARCHITECT c16000 fully automated biochemical immunoassay system) during the study period, patients were divided into the BNP elevation group (BNP > 100 pg/mL) and the non-elevation group (BNP ≤ 100 pg/mL).

### 2.3. Included indicators

Structured follow-up was conducted at 3 months, 1 year, and 2 years after aneurysm clipping or endovascular treatment. Follow-up was performed through outpatient visits and telephone interviews. The primary outcome events were defined as long-term MACE,^[18]^ including myocardial infarction,^[19]^ acute heart failure,^[20]^ arrhythmias,^[21]^ and cardiac arrest,^[22]^ as well as adverse neurological functional prognosis (mRS score of 3–6). Long-term follow-up was defined as the last recorded follow-up before patient loss to follow-up after discharge. Secondary outcome events included complications during hospitalization, such as MACE, DCI,^[23]^ hydrocephalus, seizures, intracranial infections, pneumonia, and deep venous thrombosis.^[24]^

### 2.4. Statistical analysis

Statistical analysis was performed using SPSS 26.0 software (IBM, Armonk). Normally distributed continuous variables were presented as mean ± standard deviation, and between-group comparisons were conducted using independent sample *t* tests. Categorical variables were expressed as frequencies (percentages) and analyzed using chi-square tests or Fisher’s exact probability test. Non-normally distributed variables were described as median with interquartile range, and the nonparametric Mann–Whitney *U* test was applied. To minimize data bias and confounding factors, propensity score matching (PSM) was conducted for variables with statistical differences in baseline characteristics. The study population was divided into a BNP elevation group and a BNP non-elevation group.^[25]^ PSM was performed using the PS matching module in SPSS 26.0 software, with a caliper value set at 0.02. Baseline characteristics, imaging data, perioperative variables, and follow-up information were compared between the 2 matched groups. Logistic regression analysis was conducted to adjust for statistically significant parameters and investigate the independent predictive role of elevated BNP on various outcome measures in patients with aneurysmal subarachnoid hemorrhage. 2-tailed tests were used with a significance level set at α = 0.05. A *P*-value < .05 (2-tailed) was considered statistically significant.

## 3. Results

During the period from January 2016 to December 2017, a total of 213 cases of aSAH that met the inclusion criteria were enrolled. Based on the elevation of BNP levels, the cases were divided into 2 groups: the BNP elevation group (n = 88, 41.3%) and the non-elevation group (n = 125, 58.7%). Table 1 summarizes the baseline characteristics of the 213 included aSAH patients. The mean age was 57.4 ± 12.1 years, with females accounting for 61.5%. The average follow-up period was 34.3 months. The most common medical history in this population was hypertension, and the most prevalent cardiac condition was coronary heart disease.

**Table 1:** Baseline characteristics of patients in the elevated and non-elevated BNP groups.

Variables	Overall (%) n = 213	BNP elevation (%) n = 88		BNP non-elevation (%) n = 125	*P*
Ages, yr	57.4 ± 12.1		60.7 ± 11.9	55.2 ± 11.8	.001
Female	131 (61.5)		64 (72.7)	67 (53.6)	.006
Current smoking	71 (33.3)		20 (22.7)	51 (40.8)	.008
Current alcohol	65 (30.5)		19 (21.6)	46 (36.8)	.023
Premorbid					
Diabetes mellitus	17 (8.0)		9 (10.2)	8 (6.4)	.442
Hypertension	131 (61.5)		56 (63.6)	75 (60.0)	.668
Dyslipidemia	27 (12.7)		11 (12.5)	16 (12.8)	1.000
Stroke	36 (16.9)		16 (18.2)	20 (16.0)	.713
Heart diseases					
CAD	33 (15.5)		21 (23.9)	12 (9.6)	.007
Heart failure	2 (0.9)		1 (1.1)	1 (0.8)	1.000
Arrhythmia	5 (2.3)		3 (3.4)	2 (1.6)	.406
History of brain surgery	1 (0.5)		1 (1.1)	0 (0)	.413
History of heart surgery	9 (4.2)		5 (5.7)	4 (3.2)	.493
Hunt-hess scale					.021
1–2	152 (71.4)		55 (62.5)	97 (77.6)	
3–5	61 (28.6)		33 (37.5)	28 (22.4)	
Fisher scale					.888
1–2	123 (57.7)		50 (56.8)	73 (58.4)	
3–4	90 (42.3)		38 (43.2)	52 (41.6)	
Intraventricular hemorrhage	121 (56.8)		52 (59.1)	69 (55.2)	.578
Treatment modality					.459
Microsurgical clipping	106 (49.8)		41 (46.6)	65 (52.0)	
Endovascular coiling	89 (41.8)		41 (46.6)	48 (38.4)	
Medication	18 (8.4)		6 (6.8)	12 (9.6)	

BNP = brain natriuretic peptide, CAD = coronary artery disease.

In the comparison between the 2 groups, patients with BNP elevation were older (60.7 ± 11.9 years vs 55.2 ± 11.8 years, *P* = .001), had a higher proportion of females (72.7% vs 53.6%, *P* = .006), and had fewer smokers and drinkers (22.7% vs 40.8%, *P* = .008; 21.6% vs 36.8%, *P* = .023). The BNP elevation group also had a higher prevalence of coronary heart disease (23.9% vs 9.6%, *P* = .007) and higher hunt-hess grades (37.5% vs 22.4%, *P* = .021). There were no statistically significant differences in other demographic and radiographic characteristics between the BNP elevation and non-elevation groups.

To reduce bias and the impact of confounding factors, PSM was performed for the variables with statistically significant differences mentioned above. A total of 75 pairs (59 exact matches and 16 fuzzy matches) were obtained, with 59 pairs in the BNP elevation group and 59 pairs in the non-elevation group. The summarized clinical outcomes data are available in Table S1, Supplemental Digital Content, https://links.lww.com/MD/Q252. The most common in-hospital complications after PSM were pneumonia and deep venous thrombosis. Among patients who experienced MACEs during hospitalization, the most frequent cardiac events were acute heart failure, arrhythmias, and myocardial infarction. Additionally, we found a higher incidence of myocardial infarction (13.3% vs 4.0%, *P* = .001), acute heart failure (25.3% vs 4.0%, *P* = .042), DCI (25.3% vs 10.7%, *P* = .032), and hydrocephalus (13.3% vs 1.3%, *P* = .009) in the BNP elevation group.

After PSM, there were no statistically significant differences in intracranial infection, deep venous thrombosis, and mRS > 2 at discharge. During the follow-up period after discharge, 7 patients experienced MACEs. There were no statistically significant differences in MACEs between the BNP elevation group and the non-elevation group. The difference in long-term follow-up mRS > 2 was not statistically significant (*P* > .05). Logistic regression analysis showed that elevated BNP upon admission was an independent risk factor for myocardial infarction, heart failure, in-hospital MACEs, DCI, and hydrocephalus, but not for unfavorable neurological outcomes or long-term MACEs (Table 2).

**Table 2:** Logistics regression analysis of admission BNP elevation in aneurysmal subarachnoid hemorrhage observation indicators.

Variables	Significance	OR	95% CI lower	95% CI upper
In-hospital MACE	**0.001**	4.029	1.753	9.262
Myocardial infarction	**0.038**	4.405	1.085	17.886
Heart failure	**0.002**	8.420	2.192	32.341
Arrhythmia	0.245	1.834	0.660	5.096
Cardiac arrest	0.996	–	–	–
DCI	**0.013**	3.449	1.299	9.155
Hydrocephalus	**0.020**	13.726	1.501	125.567
Seizure	0.168	5.116	0.502	52.119
Intracranial infection	0.227	0.231	0.021	2.494
Pneumonia	0.699	1.166	0.535	2.540
Deep vein thrombosis	0.171	1.721	0.791	3.742
Discharge mRS	0.419	1.395	0.622	3.128
Long-term mRS > 2	0.359	0.649	0.257	1.636
Long-term MACE	0.931	0.932	0.192	4.531

Bold values indicate results considered statistically significant (*P* < .05). BNP = brain natriuretic peptide, CI = confidential interval, DCI = delayed cerebral ischemia, DVT = deep vein thrombosis, MACE = major adverse cardiac events, mRS = modified Rankin Scale, OR = odd ratio.

## 4. Discussion

This study enrolled consecutively admitted patients with aSAH 3 days after rupture to investigate the brain-heart interaction following acute cerebrovascular events. The main findings of this study indicate that the initial abnormal BNP levels upon admission are associated with MACE during hospitalization, particularly myocardial infarction, acute heart failure, DCI, and hydrocephalus. These results provide valuable information for the daily monitoring and nursing care in emergency and intensive care units, assisting in determining whether patients with elevated BNP levels are at risk of future unexpected cardiac events during hospitalization. The acute phase of aSAH itself has a high mortality rate, and the potential life-threatening complications that follow should not be overlooked.

Natriuretic peptides are classified into 3 types: atrial natriuretic peptide (ANP) secreted by the right atrium, BNP primarily secreted by the ventricles, and C-type natriuretic peptide induced by inflammation. In 2004, Tsubokawa et al suggested that BNP, rather than ANP, is the main factor leading to poor neurological functional status after aSAH, shifting the focus of research from ANP to BNP.^[26]^ BNP is a peptide consisting of 32 amino acids, primarily produced by myocardial cells and the hypothalamus, and released when myocardial cells are stretched due to increased filling pressure. This biomarker is mainly used to detect left ventricular dysfunction and evaluate the effectiveness of treatment for congestive heart failure. In the early stage following SAH, elevated BNP levels are associated with myocardial necrosis, pulmonary edema, as well as impaired left ventricular systolic and diastolic functions.^[27]^ Its plasma levels increase rapidly and return to baseline within 1 to 2 weeks. In aSAH patients, plasma BNP levels are 2 to 3 times higher than in cerebrospinal fluid, suggesting a non-brain origin of BNP. As an effective vasodilator, BNP can directly dilate blood vessels or inhibit vasoconstrictive mediators such as endothelin. Furthermore, BNP is associated with hyponatremia due to its natriuretic and salt-wasting effects, leading to the occurrence of symptomatic cerebral vasospasm. However, the exact mechanism of BNP’s involvement in cerebral vasospasm remains unclear.

Approximately 31% to 48% of patients with aSAH experience neurogenic cardiac injury, which is a cardiac dysfunction associated with brain neurotrauma. BNP is commonly used to assess the severity of heart failure. Literature indicates a positive correlation between BNP and cardiac enzyme changes following aneurysm rupture.^[28,29]^ Researchers have applied the generalized estimating equation method, demonstrating a significant correlation between BNP and cardiac troponin I. BNP is also significantly correlated with pleural effusion volume (*P* = .0003). Multivariate analysis reveals a significant association between BNP and unfavorable neurological outcomes (mRS > 2). Therefore, BNP can serve as a biomarker for neurocardiac injury and outcomes following aSAH.^[29]^ In our study, even after PSM, elevated BNP remains significantly associated with in-hospital myocardial infarction and acute heart failure in patients (13.3% vs 4.0%, *P* = .001; 25.3% vs 4.0%, *P* = .042), further confirming BNP as a biomarker for brain-heart axis disruption in aSAH patients. However, there was no statistically significant difference in the occurrence of MACE during follow-up (4.6% vs 5.8%, *P* = 1.000). The concept of treating both the brain and heart, proposed by Academician Jizong Zhao, is highly valued in this population and can improve prognosis.

Previous studies have shown that approximately one-third of aSAH patients experience DCI, which leads to unfavorable neurological outcomes and is a severe complication. In 2011, Pam R. Taub et al from the Heart Center at the University of California first proposed a strong and independent correlation between elevated BNP levels and cerebral infarction after aSAH, particularly in patients without severe vasospasm. This finding provides further evidence that, in addition to vasospastic cerebral infarction, there are other potential mechanisms leading to cerebral infarction. BNP can serve as an effective biomarker for non-vasospastic DCI.^[16]^ DCI is not only associated with higher baseline BNP levels (admission BNP), but also with the highest BNP level reached during hospitalization and the rate of change or increase in BNP over time.^[30]^

Interestingly, even after adjusting for confounding factors, plasma BNP levels remain significantly correlated with perioperative cerebral edema (13.3% vs 1.3%, *P* = .009). A possible hypothesis is that the occurrence of cerebral edema after aSAH affects the hypothalamus and triggers BNP secretion. This hypothesis requires further research for validation.

Elevated plasma BNP is associated with abnormal ventricular wall motion, diastolic dysfunction, pulmonary edema, and elevated levels of cardiac troponin. Fujiki et al found that patients with elevated BNP had worse discharge outcomes compared to those with normal BNP levels, as assessed by the GOS score.^[31]^ In addition to short-term mortality, a single-center, retrospective follow-up study conducted by Kishima et al, with an average follow-up period of 35 ± 31 months, indicated a correlation between advanced age, elevated BNP, high-grade HH scores, and long-term mortality following aSAH. Moreover, using the AUC method, they identified the optimal cutoff value (96.6 pg/mL) for dividing patients into high and low BNP groups, revealing a higher mortality rate in the high BNP group during follow-up.^[32]^

This study innovatively compared baseline information of all patients within the cohort and then adjusted for variables with baseline differences (gender, smoking, alcohol consumption, history of coronary heart disease, hunt-hess severity score) using PSM. Particularly, the adjustment for the influence of preexisting coronary heart disease on BNP values rendered all variables no longer statistically significant, thus demonstrating greater statistical power compared to previous studies that applied time-based correction methods.^[30]^

However, this study still has certain limitations. Firstly, it is a retrospective study, and although the number of cases is larger compared to previous studies, further evaluation is necessary through sample size calculation. Secondly, there is some variability in the time of BNP collection after the onset of the disease, and confounding factors related to this timing may impact the results. Finally, although preliminary studies suggest no significant impact of intervention methods on BNP release, with the rapid advancement of endovascular interventions and microsurgical procedures, the effects of different treatment approaches on post-aSAH BNP levels and cardiac events still require further exploration.

Elevated BNP after the rupture of intracranial aneurysms is significantly associated with MACE during hospitalization, DCI, and hydrocephalus. However, elevated BNP is not related to the neurological functional status at discharge or during long-term follow-up, nor does it predict MACE. While BNP can serve as an additional basis for risk stratification during the perioperative period in patients with aneurysmal subarachnoid hemorrhage, further research is needed to explore the application of cardiac enzyme markers in the clinical management of aSAH, as well as to enhance bedside care and neurologic monitoring to improve perioperative status and neurological functional prognosis for patients.

## Acknowledgments

We thank all the staff and participants for their contribution to this study.

## Author contributions

**Conceptualization:** Aiying Guo.

**Data curation:** Aiying Guo, Cuijie Ye.

**Formal analysis:** Aiying Guo.

**Funding acquisition:** Wei Cao.

**Investigation:** Cuijie Ye.

**Methodology:** Cuijie Ye, Jing Li.

**Project administration:** Cuijie Ye, Jing Li.

**Resources:** Jing Li.

**Software:** Jing Li.

**Supervision:** Wei Cao.

**Validation:** Wei Cao.

**Visualization:** Wei Cao.

**Writing – original draft:** Aiying Guo, Cuijie Ye, Jing Li, Wei Cao.

**Writing – review & editing:** Aiying Guo, Wei Cao.

## Supplementary Material


