# Supplementary material for: Clinical application research of brain natriuretic peptide in patients with aneurysmal subarachnoid hemorrhage
Source: Medicine (Baltimore). 2025 Oct 10;104(41):e44985. doi: 10.1097/MD.0000000000044985 (PMC12517807; doi:10.1097/MD.0000000000044985)
Supplement: Supplementary file 1 [file medi-104-e44985-s001.docx]

**Clinical Application Research of Brain Natriuretic Peptide in Patients with Aneurysmal Subarachnoid Hemorrhage**

Supplementary table Outcomes between patients with and without elevated BNP levels after PSM

| Variables | Before PSM | | | | After PSM | | |
| --- | --- | --- | --- | --- | --- | --- | --- |
|  | BNP elevation=88 | | BNP non-elevation  n=125 | P | BNP elevation  N=75 | BNP non-elevation  N=75 | P |
| in-hospital MACE | | 39 (44.3) | 22 (17.6) | 0.000 | 32 (42.7) | 12 (16.0) | 0.001 |
| myocardial infarction | | 13 (14.8) | 6 (4.8) | 0.015 | 10 (13.3) | 3 (4.0) | 0.042 |
| heart failure | | 22 (25.0) | 8 (6.4) | <0.001 | 19 (25.3) | 3 (4.0) | <0.001 |
| arrhythmia | | 15 (17.0) | 12 (9.6) | 0.143 | 13 (13.3) | 7 (9.3) | 0.229 |
| cardiac arrest | | 0 (0) | 2 (1.6) | 0.513 | 0 (0) | 2 (2.7) | 0.497 |
| delayed cerebral ischemia | | 23 (26.1) | 16 (12.8) | 0.019 | 19 (25.3) | 8 (10.7) | 0.032 |
| hydrocephalus | | 11 (12.5) | 4 (3.2) | 0.013 | 10 (13.3) | 1 (1.3) | 0.009 |
| seizure | | 5 (5.7) | 1 (0.8) | 0.084 | 4 (5.3) | 1 (1.3) | 0.367 |
| intracranial infection | | 2 (2.3) | 14 (11.2) | 0.017 | 1 (1.3) | 4 (5.3) | 0.367 |
| pneumonia | | 34 (38.6) | 38 (30.4) | 0.240 | 27 (36.0) | 22 (29.3) | 0.486 |
| Deep vein thrombosis | | 36 (40.9) | 29 (23.2) | 0.007 | 28 (37.3) | 19 (25.3) | 0.159 |
| discharge mRS>2 | | 37 (42.0) | 35 (28.0) | 0.040 | 28 (37.3) | 22 (29.3) | 0.387 |
| long-term mRS>2 | | 30 (34.1) | 29 (23.2) | 0.089 | 23 (30.7) | 19 (25.3) | 0.586 |
| long-term MACE | | 3 (4.0) | 4 (3.6) | 1.000 | 3 (4.6) | 4 (5.8) | 1.000 |

*BNP: brain natriuretic peptide; mRS:modified Rankin Scale; PSM: Propensity score matching; MACE: Major adverse cardiac events
